# Predictors of mortality among adult people living with HIV/AIDS on antiretroviral therapy at Suhul Hospital, Tigrai, Northern Ethiopia: a retrospective follow-up study

**DOI:** 10.1186/s41043-019-0194-0

**Published:** 2019-11-29

**Authors:** Kebede Haile Misgina, Meresa Gebremedhin Weldu, Tewodros Haile Gebremariam, Negassie Berhe Weledehaweria, Haileslasie Berhane Alema, Yosef Sibhatu Gebregiorgis, Yonas Girma Tilahun

**Affiliations:** 1 College of Health Sciences, Aksum University, P.O. Box 1010, Aksum, Ethiopia; 2 College of Health Science, Addis Ababa University, Addis Ababa, Ethiopia; 3 Center of International Reproductive Health Training (CIRHT), Bahir Dar University, Bahir Dar, Ethiopia

**Keywords:** HIV/AIDS, ART, Mortality, Tigrai, Ethiopia

## Abstract

**Background:**

Ethiopia is striving to achieve a goal of “zero human immune deficiency virus/acquired immune deficiency syndrome (HIV/AIDS)-related deaths.” However, little has been documented on the factors that hamper the progress towards achieving this goal. Therefore, the ultimate aim of this study was to determine predictors of mortality among adult people living with HIV/AIDS on antiretroviral therapy (ART).

**Methods:**

A retrospective follow-up study was employed on all adult HIV/AIDS patients who started ART between January 1 and December 30, 2010, at Suhul Hospital, Tigrai Region, Northern Ethiopia. Data were collected by trained fourth-year Public Health students using a checklist. Finally, the collected data were entered into SPSS version 16. Then after, Kaplan-Meier curves were used to estimate survival probability, the log-rank test was used for comparing the survival status, and Cox proportional hazards model were applied to determine predictors of mortality.

**Results:**

The median follow-up period was 51 months (ranging between 1 and 60 months, inter-quartile range (IQR) = 14 months). At the end of follow-up, 37 (12.5%) patients were dead. The majority of these cumulative deaths, 19 (51.4%) and 29 (78.4%), occurred within 3 and 4 years of ART initiation respectively. Consuming alcohol (adjusted hazard ratio (AHR) = 2.23, 95% CI = 1.15, 4.32), low body weight (AHR = 2.38, 95% CI = 1.03, 5.54), presence of opportunistic infections (AHR = 2.18, 95% CI = 1.09, 4.37), advanced WHO clinical stage (AHR = 2.75, 95% CI = 1.36, 5.58), and not receiving isoniazid prophylactic therapy (AHR = 3.00, 95% CI = 1.33, 6.74) were found to be independent predictors of mortality.

**Conclusion:**

The overall mortality was very high. Baseline alcohol consumption, low body weight, advanced WHO clinical stage, the presence of opportunistic infections, and not receiving isoniazid prophylactic therapy were predictors of mortality. Strengthening behavioral and nutritional counseling with close clinical follow-up shall be given much more emphasis in the ART care and support program.

## Background

Human immune deficiency virus/acquired immune deficiency syndrome (HIV/AIDS) is one of the most destructive epidemics the world has ever witnessed and its impact goes beyond public health concerns. It primarily affects the productive population group and undermines the social and economic structures mainly in the developing countries [1]. It has claimed more than 34 million lives until the end of 2014. Currently, nearly 35 million people are living with HIV in the world [2, 3]. Sub-Saharan Africa is the most affected region where 25.8 million people living with HIV. This region also accounts for the overwhelming majority of HIV/AIDS-related deaths and almost for 70% of the new HIV infections that are occurring yearly worldwide [2]. Ethiopia is one of the sub-Saharan Africa countries most severely affected by the HIV/AIDS pandemic. According to 2011 Ethiopian Demographic and Health Survey (EDHS), the overall prevalence of HIV was 1.5% nationally and 1.8% in Tigrai region [4]. In Ethiopia, a total of 793,700 people were living with HIV and approximately 46,000 AIDS-related deaths were documented in 2013 [5].

The rapidly expanding access to antiretroviral therapy (ART) is changing the global HIV epidemic in momentous ways and AIDS-related mortality rates are declining rapidly. So far, the scaling up of ART has averted an estimated of 6.6 million AIDS-related deaths worldwide predominantly in low- and middle-income countries [6]. In addition to prolonging life, ART increases productivity and quality of life among people living with HIV and produces savings to the health care [7–9]. It has also a potential to significantly reduce the risk of HIV transmission and the spread of tuberculosis [6].

Several studies have been conducted on the benefit of ART and clients’ survival in Africa, including Ethiopia, and their findings have shown improvements in survival status [9–12]. Nevertheless, mortality has been high, particularly in the first few months after initiating ART for various reasons [9, 11–17]. Ethiopia is striving to achieve a goal of “zero HIV/AIDS-related deaths” since the past few years. However, little is documented and this study is particularly conducted to assess whether Ethiopia is on the right track to achieve a goal and generate valuable information on factors that hamper the progress towards achieving the goal.

Despite many studies, evidence based up to date information on mortality and predicting factors of the adult people living with HIV/AIDS on ART is lacking and it is urgently needed for prioritizing, designing, and initiating intervention programs aiming at improving survival status. The process of priority setting should start with the assessment and analysis of the situation that adult people living with HIV/AIDS on ART face in their real environment and the data were liable to variation among health institutions. Thus, this study was carried out to provide information regarding mortality and predicting factors among adult people living with HIV/AIDS on ART in their real environment.

## Methods

### Study design, setting, and participants

This institution-based retrospective study was conducted at Suhul Hospital, Tigrai region, North Ethiopia. It is located in Shire Endaselassie town, which is the capital of the northwest zone of the Tigrai regional state. The hospital provides chronic HIV care and supports services. There were a total of 1349 adult people enrolled into the HIV care and support program in the hospital until December 30, 2014. The study population was all adult people living with HIV enrolled into ART. Hence, all HIV-positive adults on care and support follow-up who had been started ART at Suhul Hospital between January 1 and December 30, 2010 were followed until December 30, 2014. Being naive for ART treatment and being greater or equal to 18 years old was part of the inclusion criteria. Pregnant mothers on prevention of mother to child transmission (PMTCT) and non-pregnant HIV-positive adults on ART with incomplete records were the exclusion criteria.

### Measurements

Data were extracted using the checklist from the available standard national medical registers, which have been adopted by the Ministry of Health (MoH) [5]. The registers include Pre-ART, ART, and follow-up registers. Pre-ART register consists of socio-demographic characteristics, living conditions, and membership of community support/HIV support groups, disclosure status, substance abuse, WHO clinical stage, CD4 count, and prophylactic therapy. The ART register contains ART eligibility criterion, ART regimen, body weight, hemoglobin level, functional status, WHO clinical stage, CD4 count, opportunistic infections (OIs), tuberculosis (TB) status, and prophylactic or other medications given. The follow-up register is a medical register completed for all patients at each visit, and on which information regarding progressive body weight change, functional status, WHO clinical stage, TB status, newly diagnosed OIs, prophylactic or other medications given, ART adherence, reason for poor adherence, ART regimen change, the reason for any ART regimen change, drug toxicity or side effect, and laboratory test results documented. Survival status and reasons for any lost to follow-up patients are also documented on the follow-up register by tracing them through HIV-positive peers. Moreover, reasons for patients transferred to another health facility are recorded in the follow-up register. The follow-up data were documented electronically for the majority of the patients in the study hospital. However, the survival details for lost to follow-up patients were not well documented.

The checklist used for recording information extracted from medical registers and the electronic database was adapted from the available standard national medical registers. The checklist included socio-demographic and behavioral characteristics, baseline clinical and immunological profiles, and survival status.

The data were collected by four graduating class public health students under close supervision of two senior public health professionals and the principal investigators. Prior to data collection, training was given to both data collectors and supervisors for 1 day on how to use the checklist and collect data from the medical registers and electronic database. The completed checklists were checked out for completeness, accuracy, consistency, and clarity on a daily basis by the supervisors just by comparing them with the medical registers. A randomly selected 10% of the completed checklists were also checked against the medical registers by the principal investigators.

The main endpoint of this study was death and its time of occurrence in any of the 5-year study period from all causes. Deaths and other outcomes, including a time when patients transferred to another health facility and lost to follow-up, were recorded from the medical registers. Patients who missed appointments for more than 3 months were considered as lost to follow-up. The rest patients who were still alive and followed-up were censored on December 30, 2014.

### Data processing and analysis

Data were entered, cleaned, and analyzed by SPSS version 16.0 statistical package for windows. The survival time was calculated in months using the time interval between the date of ART initiation and date of the event (death) for events, date of transfer for transferring out (TO), first date of the first missed appointment for lost to follow-up cases, and the date in which patient completed the follow-up time. Kaplan-Meier and Cox proportional hazards techniques were used to identify predictors of death [18–21]. Kaplan-Meier curves were used to estimate survival probability after ART initiation and log-rank tests were used to compare the survival curves. Bivariate Cox proportional hazards model was fitted for all explanatory variables. A *p* value *≤* 0.2 was set in the bivariate analysis as criteria to select candidate variables for multivariate Cox proportional hazards model. Finally, multivariate Cox proportional hazards model with the backward LR method was fitted to identify independent predictors of death. Hazard ratio at 95% confidence interval and *p* value were used to measure the strength of association and identify statistical significant result. Results were considered statistically significant when *p* value *<* 0.05.

## Results

### Baseline socio-demographic characteristics

A total of 295 patients were included in this study. The mean age and standard deviation of the study participants were 35.02 ± 9.35 years. One hundred forty-five (49.1%) were > 35 years old. More than half (54.2%) were females, three-fourth (74.9%) were from urban areas, 269 (91.2%) were Christian in religion, 153 (52.1%) were married, and nearly one-third (31.5%) had no formal education. Of the total patients included in this study, 55 (18.6%), 46 (15.6%), and 46 (15.6%) were housewives, merchants and government employees in occupation respectively. Of the total patients included in this study, 14 (4.7%), 22 (7.5%), and 86 (29.2%) were smoking tobacco, chewing khat (*Catha edulis*), and consuming alcohol respectively at the initiation of ART (Table 1).

**Table 1:** Baseline socio-demographic and behavioral characteristics of adult people living with HIV on ART at Suhul Hospital, Northern Ethiopia, 2010–2014

Characteristics	Frequency	Percent (%)
Age		
< 25	25	8.5
25–34	125	42.4
≥ 35	145	49.1
Sex		
Male	135	45.8
Female	160	54.2
Residence		
Urban	221	74.9
Rural	74	25.1
Religion		
Christian	269	91.2
Muslim	26	8.8
Marital status		
Married	153	52.1
Never married	55	18.7
Divorced	71	24.1
Widowed	15	5.1
Education		
No formal education	93	31.5
Primary	122	41.3
Secondary	49	16.6
Above secondary	31	10.5
Occupation		
Housewife	55	18.6
Merchant	46	15.6
Governmental employed	46	15.6
Farmer	29	9.8
Daily laborers	25	8.5
No job	25	8.5
Commercial sex worker (CSW)	21	7.1
Self-employed	19	6.4
Non-governmental employed	17	5.8
Others*	12	4.1
Household size		
1–4	256	86.8
5 or more	39	13.2
Smoke tobacco		
Yes	14	4.7
No	281	95.3
Consume alcohol		
Yes	86	29.2
No	209	70.8
Chew khat		
Yes	22	7.5
No	273	92.5

*Student, prisoner, and retired

### Baseline clinical and immunological profile

Around 30% of the patients had advanced clinical symptoms (WHO clinical stage III or stage IV) and more than half (59.0%) of the patients had advanced disease (CD4 count of 200 cells/mm^3^ or below) at the initiation of ART. Concerning to their functional status, 37 (12.5%) patients were either bedridden or ambulatory. Nearly three-fourth (74.6%) and 80 (27.1%) of the adult people living with HIV enrolled on ART were on cotrimoxazole prophylactic therapy (CPT) and isoniazid prophylactic therapy (IPT) at the initiation of ART respectively. Of the total patients included in this study, 120 (42.7%) had < 50-kg baseline body weight and 87 (29.5%) had anemia (hemoglobin level < 11 gm/dl) (Table 2).

**Table 2:** Baseline clinical characteristics of adult people living with HIV on ART at Suhul Hospital, Northern Ethiopia, 2010–2014

Characteristic	Number	Percent (%)
Weight		
< 50 kg	126	42.7
≥ 50 kg	169	57.3
Hemoglobin level (g/dl)		
< 11 g/dl	87	29.5
≥ 11 g/dl	208	70.5
CD4 count (cells/mm^3^)		
≤ 200	174	59.0
> 200	121	41.0
WHO clinical stage		
I	95	32.2
II	113	37.3
III	65	22.0
IV	22	7.5
Functional status		
Working	258	87.5
Ambulatory	31	10.5
Bedridden	6	2.0
Opportunistic infections (OIs)		
Yes	88	29.8
No	207	70.2
Tuberculosis disease status		
Absent	269	91.2
Present	26	8.8
Cotrimoxazole prophylactic therapy (CPT)		
Yes	220	74.6
No	75	25.4
Isoniazid prophylactic therapy (IPT)		
Yes	80	27.1
No	215	72.9
Fluconazole prophylactic therapy		
Yes	9	3.1
No	286	96.9
Eligibility criteria for initiating ART		
Clinically	59	20.0
Immunologically	157	53.4
Both	79	26.8
Type of ART initiated		
AZT-3TC-NVP	145	49.2
d4T(30)-3TC-NVP	109	37.0
d4T(30)-3TC-EFV	33	11.2
d4T(40)-3TC-EFV	3	1.0
d4T(40)-3TC-NVP	5	1.7
Disclosed HIV serostatus		
Yes	237	80.3
No	58	19.7
HIV serostatus disclosed to (*n* = 237)		
Wife/husband	140	47.5
Brother/sister	41	13.9
Own children	39	13.2
Parent	4	1.4
Friends	13	4.4

More than half of the patients (53.4%) were enrolled in ART based on immunological eligibility criteria while the rest were enrolled either based on clinical eligibility criteria or based on both clinical and immunological criteria. AZT-3TC-NVP (49.2%) and d4T-3TC-NVP [22] (37.0%) were the most prescribed ART drugs at the ART initiation. Concerning to disclosing HIV serostatus, a total 237 (80.3%) of the patients disclosed their serostatus either to their spouse, sibling, children, friends, or parents (Table 2).

Almost 30% of the total patients included in the present study had at least one opportunistic infection (OI) at the initiation of ART. Tuberculosis was the leading OI (29.6%) followed by Zoster 21 (7.1%), diarrhea 19 (6.4%), and *Pneumocystis carinii* pneumonia (PCP) 17 (5.8%) (Table 3).

**Table 3:** Opportunistic illnesses among adult people living with HIV at initiation of ART at Suhul Hospital, Northern Ethiopia, 2010–2014

S. No.	Opportunistic illnesses	Frequency	Percent
1	Tuberculosis	26	8.8
2	Zoster	21	7.1
3	Diarrhea (chronic/acute)	19	6.4
4	*Pneumocystis carinii* pneumonia (PCP)	17	5.8
5	Bacterial pneumonia	4	1.4
6	Thrush (oral/genital)	4	1.4
7	Central nervous system toxoplasmosis	4	1.4
8	Cryptococcal meningitis	4	1.4
9	Ulcer (oral/genital)	2	0.7
10	Other	13	4.4

### Predictors of mortality

**Fig. 1:**
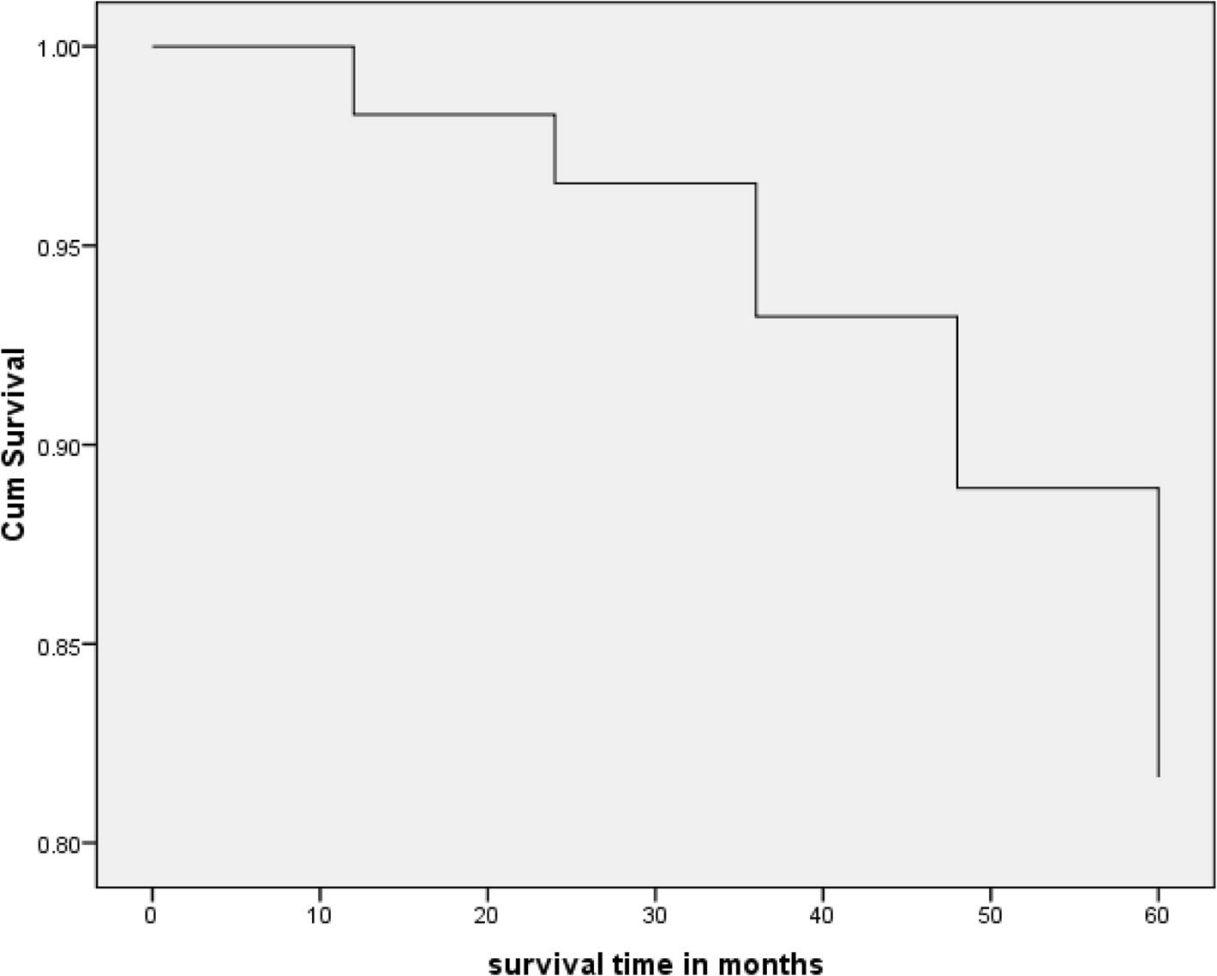
Kaplan-Meier survival curve among adult people living with HIV on ART at Suhul Hospital, Northern Ethiopia, 2010–2014

The median follow-up period was 51 months (ranging between 1 and 60 months, IQR = 14 months). At the end of follow-up, 207 (70.2%) adult people living with HIV on ART were alive, 18 (6.1%) were lost to follow-up, 33 (11.2%) were transferred out to other facilities, and 37 (12.5%) were reported to be dead. The mortality rate was 0.28 per 100 person-years of observation. Concerning the time of death, 5 (13.5%), 10 (27.0 %), 19 (51.4%), and 29 (78.4%) of the deaths occurred within the first 1, 2, 3, and 4 years of ART initiation respectively. Little information was available about the possible cause(s) of death. After starting treatment, the mean time to death was 33.03 months (95% CI 27.5, 38.6 months). The overall survival probability among adult people living with HIV on ART declined over follow-up time. The cumulative probabilities of survival at 1, 2, 3, 4, and 5 years of ART initiation were 0.98, 0.97, 0.93, 0.89, and 0.82 respectively (Fig. 1).

**Fig. 2:**
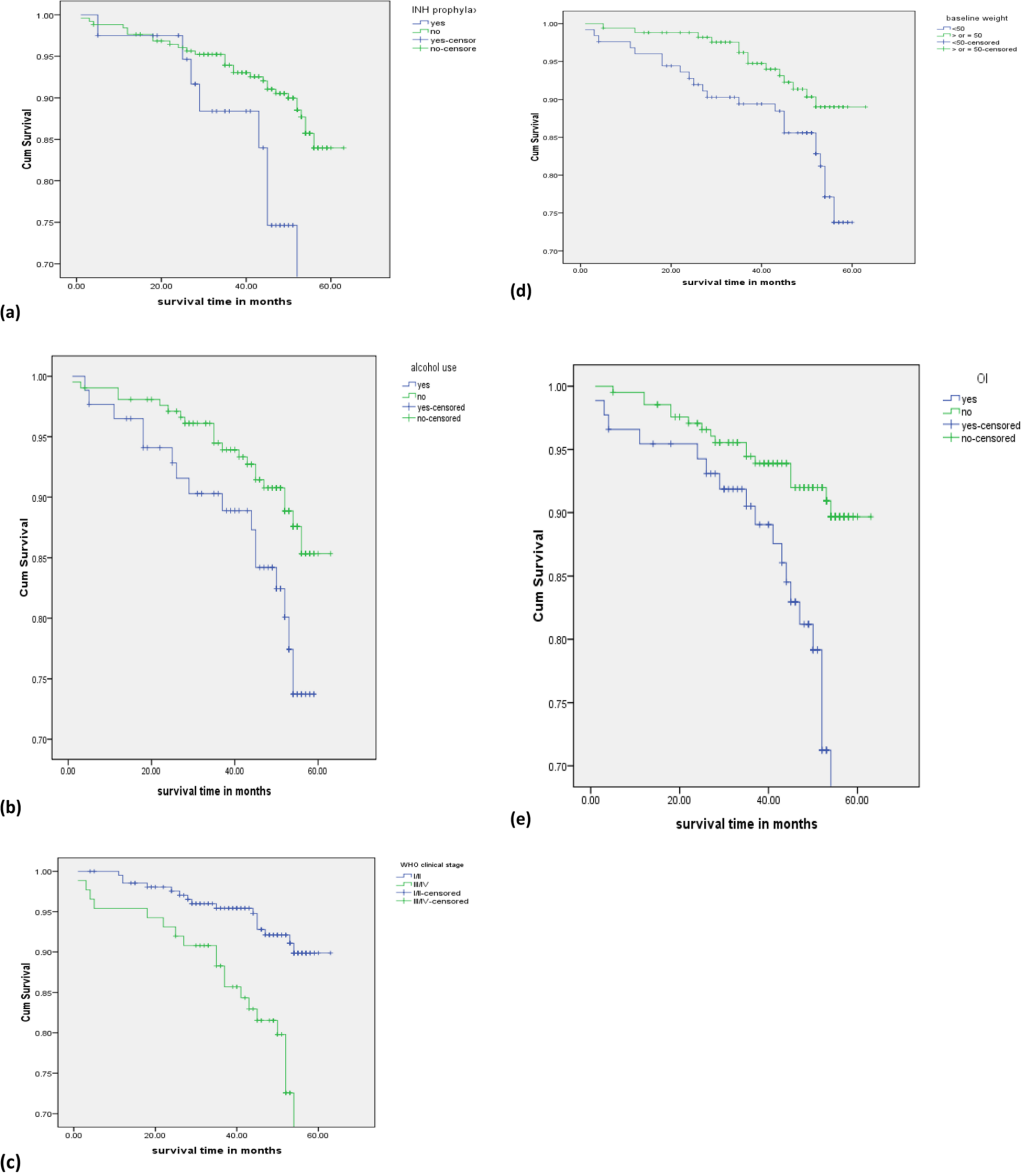
Kaplan-Meier survival functions stratified according to **a** IPT, **b** alcohol consumption, **c** WHO clinical stage, **d** body weight, and **e** OIs among adult people living with HIV on ART at Suhul Hospital, Northern Ethiopia, 2010–2014

In bivariate analysis, the results of the study revealed that marital status, alcohol consumption, khat chewing, low body weight, advanced WHO clinical stage, functional status, the presence of OIs, active tuberculosis disease, and not receiving isoniazid prophylactic therapy were associated with mortality among the various baseline factors included in the study. Figure 2 shows the Kaplan-Meier survival curves.

In the multivariate Cox regression analysis, alcohol consumption, low body weight, advanced WHO clinical stage, the presence of OIs, and not receiving isoniazid prophylactic therapy were significantly associated with mortality. Hence, consuming alcohol at baseline resulted in more than twofold risk of dying (AHR = 2.23, 95% CI = 1.15, 4.32). Patients with low baseline body weight (< 50 kg) were at more than two times increased the risk of death (AHR = 2.38, 95% CI = 1.03, 5.54) compared with their counterparts. Patients who suffered from OIs at ART initiation had more than two times risk of dying (AHR = 2.18, 95% CI = 1.09, 4.37) than those who were free of OIs. Similarly, patients with advanced WHO clinical stage (III and IV) at the initiation of ART were more than two times at risk of dying (AHR = 2.75, 95% CI = 1.36, 5.58) than those who were with less advanced WHO clinical stage (I and II). Furthermore, patients who did not receive isoniazid prophylactic therapy were three times at higher risk of death (AHR = 3.00, 95% CI = 1.33, 6.74) compared with those who received isoniazid prophylactic therapy (Table 4).

**Table 4:** Multivariate Cox regression analysis to determine predictors of mortality among adult people living with HIV on ART at Suhul Hospital, Northern Ethiopia, 2010–2014

Characteristics	Survival status		Crude HR (95% CI)	Adjusted HR (95% CI)
	Died	Censored		
Age				
< 25	7	18	2.28 (0.96,5.42)	
25–34	11	114	0.70 (0.34,1.48)	
≥ 35	19	126	1	
Marital status				
Married	13	141	1	
Not married	11	44	2.43 (1.09, 5.41)	
Divorced/widowed	13	73	1.80 (0.84, 3.89)	
Alcohol consumption				
No	21	188	1	1
Yes	16	70	2.02 (1.06, 3.88)	2.23 (1.15, 4.32)
Chat chewing				
No	6	16	1	
Yes	31	242	2.61 (1.09, 6.26)	
Weight				
< 50 kg	23	103	2.13 (1.09, 4.14)	2.38 (1.03,5.54)
≥ 50 kg	14	155	1	1
Opportunistic infections				
No	17	190	1	1
Yes	20	68	3.20 (1.68, 6.13)	2.18 (1.09, 4.37)
WHO clinical stage				
I/II	16	192	1	1
III/IV	21	66	3.47 (1.81, 6.68)	2.75 (1.36,5.58)
Functional status				
Working	26	232	1	
Ambulatory/bed ridden	11	26	3.28 (1.62,6.64)	
Isoniazid prophylactic therapy (IPT)				
No	29	186	2.65 (1.19, 5.87)	3.00 (1.33, 6.74)
Yes	8	72	1	1
Tuberculosis disease status				
Absent	29	240	1	
Present	8	18	3.22 (1.47, 7.05)	

## Discussion

This retrospective follow-up study gives an insight into mortality and its predictors among adult people living with HIV/AIDS enrolled on ART. In this study, a total of 37 (12.5%) deaths occurred during the follow-up period. This was similar to studies in Ethiopia, Uganda, Nepal, and South Africa [23–30]. Regarding the mortality time, 5 (13.5%), 10 (27.0 %), 19 (51.4%), and 29 (78.4%) of the deaths occurred within the first, second, third, and fourth years of ART initiation respectively. The mean time to death was 33.03 months (95% CI 27.5, 38.6 months). Though the overall mortality was still very high, the mortality in the first year is lower compared with other studies in Ethiopia and other developing countries [11, 22, 23, 28, 30, 31], in which it was found to be within the range of 22.9% to 92.6%. However, a finding of the current study is still similar to the finding of a recent meta-analysis [32], in which the pooled estimated 1-year probability of death from studies conducted in Africa was 17% (95% CI 11 to 24%) [32]. The implication is that the current progress of reducing HIV/AIDS-related death is not promising to achieve the goal of “zero HIV/AIDS-related death” in the near future.

Moreover, there was a high loss to follow-up of patients in the present study, which is similar to the finding of a study done by Bucciardini et al. [33]. The main reasons documented by tracing the lost to follow-up patients include migration to another area for searching work, coming from another area which is far from the hospital fearing stigma and lack of money for transportation. The other interesting point is that the survival status of the lost to follow-up patients may be different from the worst-case scenario (death) because their clinical characteristics were similar to those who were alive at the end of the follow-up. So, it is not acceptable to carry out a sensitivity analysis called “the worst-case scenario Cox proportional hazard model” in which all lost to follow-up patients were assumed as “died” immediately after their last contact with the hospital. This is a different scenario which needs tracing back when such a case happens, perhaps they may stop the treatment or they may go into traditional medicine. In addition to the high lost to follow-up, there was a high transfer to other facilities in this study. The most common reasons mentioned for transfer to other facilities were a family reason, search for work, and poor approach of service providers.

In this study, baseline alcohol consumption, body weight, WHO clinical stage, OIs, and not receiving isoniazid prophylactic therapy were independent predictors of mortality among HIV/AIDS patients enrolled on ART. HIV/AIDS patients who enrolled on ART with a baseline body weight of < 50 kg were more than two times at higher risk of dying. This finding is in line with several other studies conducted in Cameroon, Nepal, South Africa, Tanzania, and Ethiopia [12, 22, 28–31, 34]. The possible explanation for this could be lower body weight is a proxy indicator of poor nutritional status, which weaken the immunity and favors the flourishing of OIs like TB that aggravates morbidity and speeds mortality among HIV/AIDS patients on ART [11, 12, 34].

Furthermore, the risk of mortality has been significantly higher among those who were suffering from advanced WHO clinical stage (stage III or IV) of the disease at ART initiation compared with their counterparts. This is in agreement with the findings of studies done in Nepal, Cameroon, Zambia, South Africa, and Ethiopia [7, 10, 12, 22, 23, 28, 29, 31]. This could be due to the fact that advanced clinical stage is an indication of much-weakened immunity, which results in OIs, the major causes of mortality among HIV-infected patients. However, this finding is not in line with the finding of other studies in Ethiopia, Tanzania, Botswana, and in many low-income countries [7, 11, 17, 30]. The inconsistency might be due to the difference in time of initiating ART. That is, the great majority of people living with HIV included in the previous studies initiated ART lately in the advanced clinical stage which could mask the difference. Moreover, this could be due to the difference in time the studies conducted and the duration of follow-up.

The presence of OIs among HIV/AIDS patients enrolled on ART was also another independent predictor of mortality; HIV/AIDS patients who experienced OIs were about three times at higher risk of dying compared with their counterparts. This finding is in agreement with studies conducted in many developing countries, which showed OIs such as tuberculosis, *Pneumocystis carinii* pneumonia, and toxoplasmosis as the main causes of mortality in patients with HIV infection on ART [14, 15, 27, 35, 36]. This may be due to late enrollment, lack of close follow-up of people living with HIV enrolled on ART, and management of patients with OIs promptly.

Furthermore, patients who did not receive isoniazid prophylactic therapy at the initiation of ART or before in the course of HIV/AIDS care and support have been found to be more than three times at higher risk of dying when compared with those who received IPT. This finding is supported by the study conducted in Debre Markos referral hospital, Ethiopia [24]. This could be due to the fact that isoniazid prophylactic therapy prohibits reactivation and re-infection of tuberculosis among HIV patients. It has been also proven by many studies to be one of the ultimate tuberculosis preventive strategies [15, 36]. This implies that isoniazid prophylactic therapy seems to be a more plausible alternative in the prevention of tuberculosis in the resource-limited settings such as Ethiopia.

However, there was no association between tuberculosis and mortality in this study opposite to the findings from other similar studies conducted elsewhere [25, 27, 31]. The possible reason for this might be patients who died were not under isoniazid prophylactic therapy and there is evidence that taking isoniazid prophylactic therapy delays infection from TB which is the most common killer among people living with HIV/AIDS. The other probable reason could be the prevalence of tuberculosis is lower (8.8%) in this study; despite HIV/AIDS and tuberculosis, co-infection occurs in nearly 50% of people living with HIV/AIDS in Ethiopia. So, the low prevalence of tuberculosis might mask the association.

Alcohol consumption has been found to be another significant predictor of mortality in this study. HIV/AIDS patients on ART who consumed alcohol were more than two times at higher risk of death compared with those who did not consume alcohol. This lends support to recent findings of systematic review study done by Azar et al [37–39]. This could be due to the reason that alcohol use alone can be associated with decreased ART uptake, adherence, and viral suppression [37–42]. This suggests that behavioral factors such as alcohol consumption should be given due emphasis in the HIV care and support program.

Finally, it is worth mentioning some weaknesses of this study. First, mortality might be underestimated, since patients lost to follow-up probably include individuals dying at home without being reported. In addition to this, since secondary data was used for this study, it was impossible to include some key variables such as economic status and psychological distress that need to be included in this study. There was also incompleteness of records for some of the patients enrolled on ART. On the plus side, because the study was done during the time when Ethiopia is striving to achieve its recently planned “zero HIV/AIDS-related goal,” the findings may give better insights into the problems that shall be considered to achieve the goal.

## Conclusion

In conclusion, the findings of this study point out that there was still high mortality. The final Cox proportional hazards model identified alcohol consumption, low baseline body weight, advanced WHO clinical stage, opportunistic infections, and not receiving isoniazid prophylactic therapy as predictors of mortality. Hence, early initiation of ART, isoniazid prophylactic therapy, and close clinical follow-up with behavioral and nutritional counseling and support as well should be given due emphasis on the ART care and support program. Further study that explores on the clients who lost to follow-up and methods of tracing is recommended.

## Data Availability

All the data supporting the findings is contained within the manuscript, no additional data are needed.

## Abbreviations

AHR: Adjusted hazard ratio
ART: Antiretroviral therapy
CI: Confidence interval
CPT: Cotrimoxazole prophylactic therapy
EDHS: Ethiopian Demographic and Health Survey
HIV/AIDS: Human immune deficiency virus/acquired immune deficiency syndrome
IPT: Isoniazid prophylactic therapy
IQR: Inter-quartile range
OIs: Opportunistic infections
PCP: *Pneumocystis carinii* pneumonia
PMTCT: Prevention of mother to child transmission
TB: Tuberculosis
WHO: World Health Organization
